# Changes in proximal colon on intestinal ultrasound indicate the efficacy of induction therapy for active ulcerative colitis

**DOI:** 10.1007/s10396-025-01554-z

**Published:** 2025-06-16

**Authors:** Koichi Izumikawa, Tomoki Inaba, Eriko Yasutomi, Sakuma Takahashi, Hugh Shunsuke Colvin, Ichiro Sakakihara, Kumiko Yamamoto, Shigetomi Tanaka, Shigenao Ishikawa, Masaki Wato

**Affiliations:** Department of Gastroenterology, Kagawa Prefectural Central Hospital, 1-2-1 Asahi-machi, Takamatsu, 760-8557 Japan

**Keywords:** Colitis, Ulcerative, Ultrasonography, Induction therapy, Colonoscopy

## Abstract

**Purpose:**

This study aimed to elucidate the effectiveness of intestinal ultrasound (IUS) in assessing induction therapy for active ulcerative colitis (UC).

**Methods:**

Thirty-two patients (12 with severe colitis and 20 with moderate colitis) were analyzed. Disease activity was assessed using the Mayo endoscopic score (MES) obtained via colonoscopy. Ultrasound (US) grade and bowel wall thickness (BWT) before induction therapy were compared in terms of MES. Changes in US grade and BWT from pre-treatment to clinical improvement were analyzed at the most inflamed segments, oral side, and anal side.

**Results:**

A correlation coefficient of 0.58 (*p* < 0.01) indicated a strong correlation between US grade and MES. US grade and BWT decreased at the most inflamed segments, oral side, and anal side for clinical improvement in all cases (*p* < 0.01, *p* < 0.01, respectively). However, changes in US grade were significantly greater on the oral side than the most inflamed segments or the anal side (*p* = 0.008 and *p* = 0.039, respectively).

**Conclusion:**

IUS is an effective method for assessing the efficacy of induction therapy for active UC. The assessment should be performed at the proximal part of the colon, rather than solely at the site of the most pronounced inflammation.

**Supplementary Information:**

The online version contains supplementary material available at 10.1007/s10396-025-01554-z.

## Introduction

Ulcerative colitis (UC) is a diffuse, nonspecific inflammatory disease of unknown origin that primarily affects the mucosa and submucosa and commonly causes erosions and ulcers. The lesions form continuously and diffusely in the colon and rectum. Colonoscopy (CS) is the gold standard for evaluating UC activity; however, frequent use of this technique is difficult, especially during the acute phase when inflammation is severe, as it is physically invasive.

Intestinal ultrasound (IUS) requires no pretreatment, does not involve radiation exposure, is noninvasive, and is easy to perform [1, 2], making it a useful modality for evaluating the inflammatory site and disease activity of UC [3–6]. Bowel wall thickness (BWT) [7] is commonly used to evaluate the severity of UC using IUS. Furthermore, several classifications have been proposed for estimating changes in the intestinal wall structure [8, 9].

While numerous studies have explored the utility of IUS in evaluating the disease status of UC, its usefulness in determining the treatment efficacy for acute UC has not been sufficiently examined. In the treatment of severe ulcerative colitis, it is essential to properly evaluate the effectiveness of pharmacological therapy and consider surgical colectomy for life-saving purposes. However it has been reported that during the healing process of UC, 88.0% of patients show improvement from the proximal colon toward the distal colon [10]. Moreover, IUS is susceptible to variations arising from differences in equipment and settings, as well as variations among examiners [11].

This retrospective cohort study, in which IUS data were obtained by a single practitioner, was conducted to investigate the usefulness of IUS, including the appropriate assessment sites, in evaluating induction therapy.

## Materials and methods

### Patients

The study cohort comprised 39 consecutive patients diagnosed with active UC who were hospitalized and treated between January 2016 and December 2022. To evaluate disease status, CS and IUS were performed at the time of admission, and the course of UC was evaluated using IUS once a week. Exclusion criteria included severe ulcerative colitis that precluded colonoscopy, the proctitis type of UC, confirmed infectious gastroenteritis such as cytomegalovirus infection, and inability to induce clinical improvement. The study design was approved by the ethics committee of Kagawa Prefectural Central Hospital (Registry No. 1173), and adhered to the Declaration of Helsinki. Informed written consent was obtained from each participant before they were enrolled in the study.

The extent of lesions and the sites displaying the most pronounced inflammation were determined using CS. UC severity was assessed using the Mayo score [12] before induction therapy, i.e., baseline, and the most inflamed segments were determined in five segments from the cecum to the sigmoid colon. The oral and anal segments of the most inflamed segments were defined as the oral side and the anal side, respectively. In instances where the assessment of the entire colon using CS was difficult, the extent of the lesion was diagnosed by assessing colon wall thickening using computed tomography (CT). CS was performed after IUS, irrespective of whether CS pretreatment had been administered. Regarding the assessment of efficacy following therapeutic intervention, the partial Mayo score (PMS) [13] was concurrently evaluated with IUS, and clinical improvement was defined as a reduction of two or more points in the PMS [14].

US grade and BWT before induction therapy were compared with the Mayo endoscopic score (MES) findings. In addition, a comparison was made between US grade and BWT at baseline and at clinical improvement to determine the usefulness of our US classification.

The primary endpoints were the effectiveness and optimal methodology of IUS in assessing induction therapy. Therefore, the changes in US grade and BWT from pre-treatment to clinical improvement in the most inflamed segments were compared with those on the oral or anal side.

### IUS

IUS evaluations were performed by a single specialist utilizing the Aplio 500 (Cannon Medical Systems Corp., Otawara, Japan), primarily by using a linear probe (7.5 MHz), while a convex probe (3.5–6.0 MHz) was used to evaluate the deep intestinal tract. IUS was performed upon admission and once weekly thereafter. UC severity was assessed using the US grade classification and BWT.

The US grade classification was developed by modifying the classification reported by Hata et al. [8] (Supplementary Fig. 1). The US grade classification was divided into five stages based on the state of intestinal wall thickening and layered structure (which reflect inflammation) using cross-sectional diagnostic methods. US grade 0 was defined as no wall thickening, indicating the absence of inflammation, and presence of a normal layer structure. US grade 1 was defined as mild wall and second-layer thickening, indicating mucosal inflammation. US grade 2 was defined as thickening of the second and third layers without hypoechoic changes in the third layer, indicating that the inflammation had spread to the submucosa. US grade 3 was defined as thickening of the second and third layers with hypoechogenicity of the third layer. US grade 4 was defined as wall thickening with an unclear full-thickness structure, representing severe full-thickness inflammation.

The vertical distance from the mucosal border echo to the serosal border echo within the intestinal wall was measured at three different locations in the longitudinal section for each segment (Supplementary Fig. 2) and the resultant average value was used as the BWT at each segment. The ability to visualize the entire rectum with IUS is low [9, 15]. Therefore, we evaluated six segments consisting of the cecum, ascending colon, transverse colon, descending colon, sigmoid colon, and rectal-sigmoid colon (Rs).

### Statistical analysis

The relationships between the US grade and MES or BWT before induction therapy were evaluated using Pearson’s correlation coefficient. The relationship between BWT and MES was analyzed using Student’s or Welch’s t-test (significance level adjusted by Bonferroni multiple comparisons, α = 0.0083). Comparisons of the US grade and BWT at baseline and at clinical improvement in the most inflamed segments, oral side, and anal side, were analyzed using a paired t-test. Comparisons of the changes in each parameter between sites were performed using one-way analysis of variance and the Bonferroni method.

## Results

### Patients

Seven of the 39 enrolled patients were excluded: three had complications of cytomegalovirus infection, two were unable to undergo CS, and the treatment was ineffective for two. No patient had proctitis Therefore, the final analysis of the patients with clinical improvement included 32 patients (Table 1).

**Table 1:** Patient characteristics

Patients	*n* = 32
Age, years	40 (13–83)
Sex: Male/Female	20/12
Disease location: Pancolitis/Left-sided	31/1
Disease duration, years	4.5 (0–27)
Disease severity: Severe/Moderate	12/20
Mayo score	10 (6–12)
Partial Mayo score	7(3–9)
Hospitalization, days	20 (14–28)
IUS examination, times	3 (2–4)

The patient cohort comprised 20 males and 12 females, with a median age of 40 years (range, 13–83). Thirty-one had pancolitis and one had left-sided colitis; and the median duration of UC was 4.5 years (range, 0–27). The number of UC exacerbations requiring hospitalization was 25 in the first attack, two in the second, four in the third, and one in the fourth. The median Mayo score on admission was 10 (range, 6–12), with 12 and 20 patients categorized with severe colitis and moderate colitis, respectively. The median PMS score at admission was 7 (range, 3–9).

The entire colon was observed during the initial CS in 14 of the 32 patients (43.8%). However, owing to the patients’ serious condition, the sigmoid colon was observed in eight, descending colon in two, transverse colon in seven, and ascending colon in one. Conversely, it was possible to evaluate the entire colon except the lower rectum using IUS in all patients. Regarding the extent of the lesion diagnosed using CT, 31 patients were diagnosed with pancolitis and one with left-sided colitis; these outcomes were consistent with those of IUS.

**Fig. 1:**
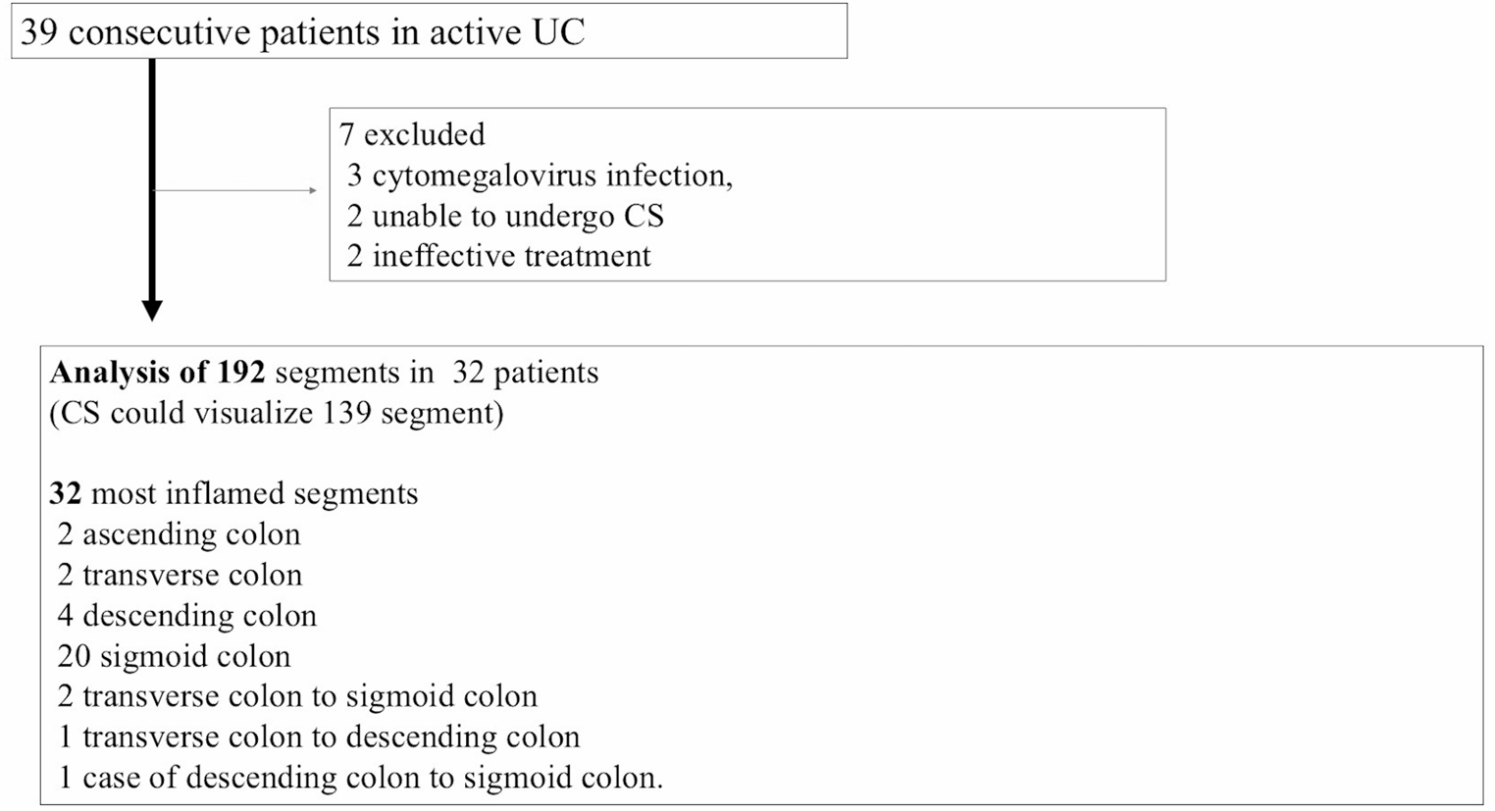
Flow diagram of selection of patients with active UC on admission

The most inflamed segments in 32 patients determined based on CS were two in the ascending colon, two in the transverse colon, four in the descending colon, 20 in the sigmoid colon, two in the transverse colon to sigmoid colon, one in the f transverse colon to descending colon, and one in the descending colon to sigmoid colon. Figure 1 shows a flow diagram of the patient selection process.

Induction therapy consisted of prednisolone in 20 patients, tacrolimus in 12, cytapheresis in nine, infliximab and adalimumab in two, and vedolizumab and golimumab in one, including duplicate treatments.

The median length of hospitalization was 20 days (range, 8–69), and the IUS procedure was performed three times (median) during that period (range, 2–9) (Table 1).

### Comparison among US grade, BWT, and MES before induction therapy

The median of US grades and BWT in 32 patients before induction therapy were 3 (2–4) and 6.3 (3.9–9.5), respectively.

**Fig. 2:**
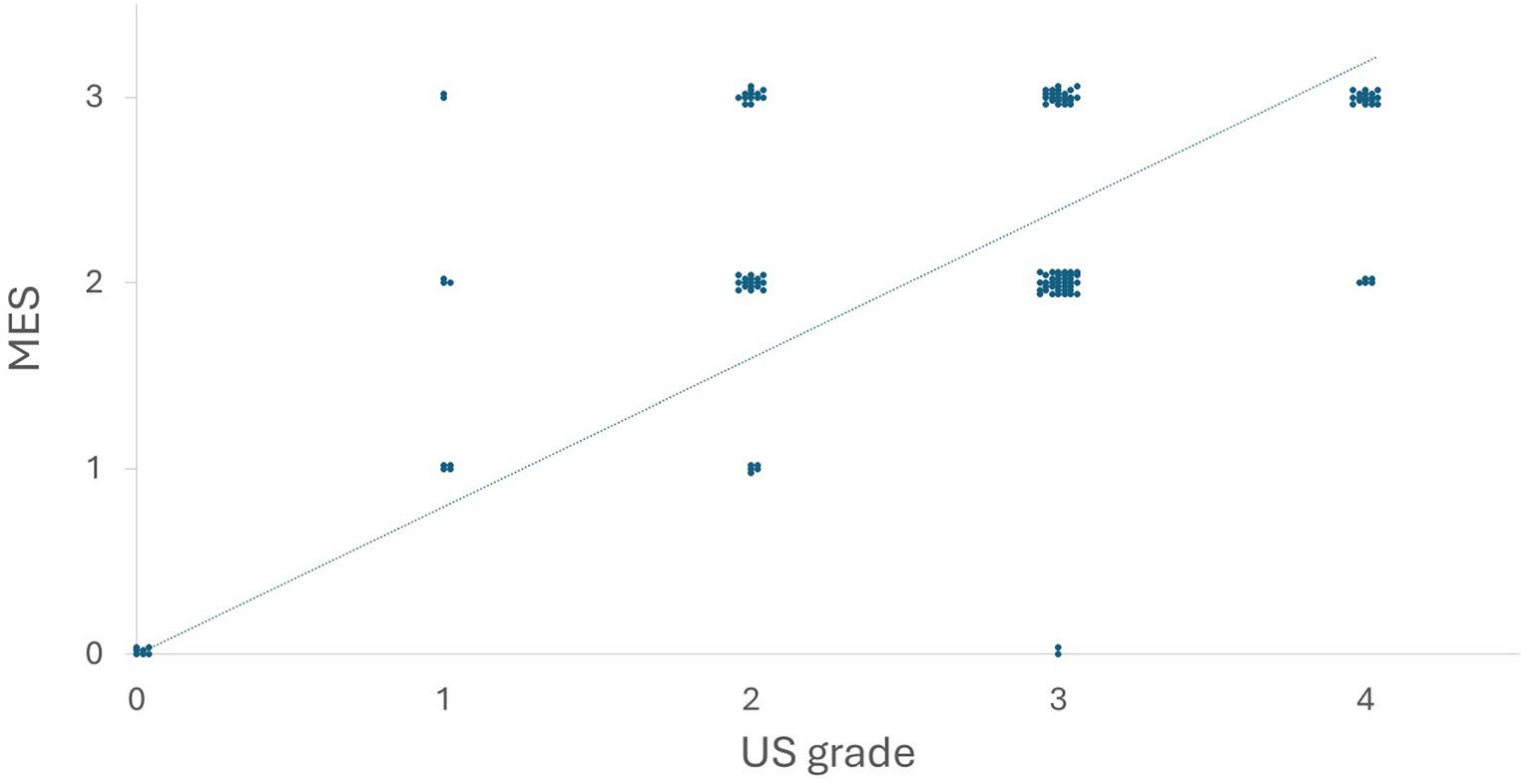
Relationship between US grade and MES. MES, Mayo endoscopic score

The median US grades for endoscopically active MES1, MES2, and MES3 on admission were 2, 3, and 3, respectively. A correlation was found between US grade and MES (*r* = 0.58, *p* < 0.01) (Fig. 2).

**Fig. 3:**
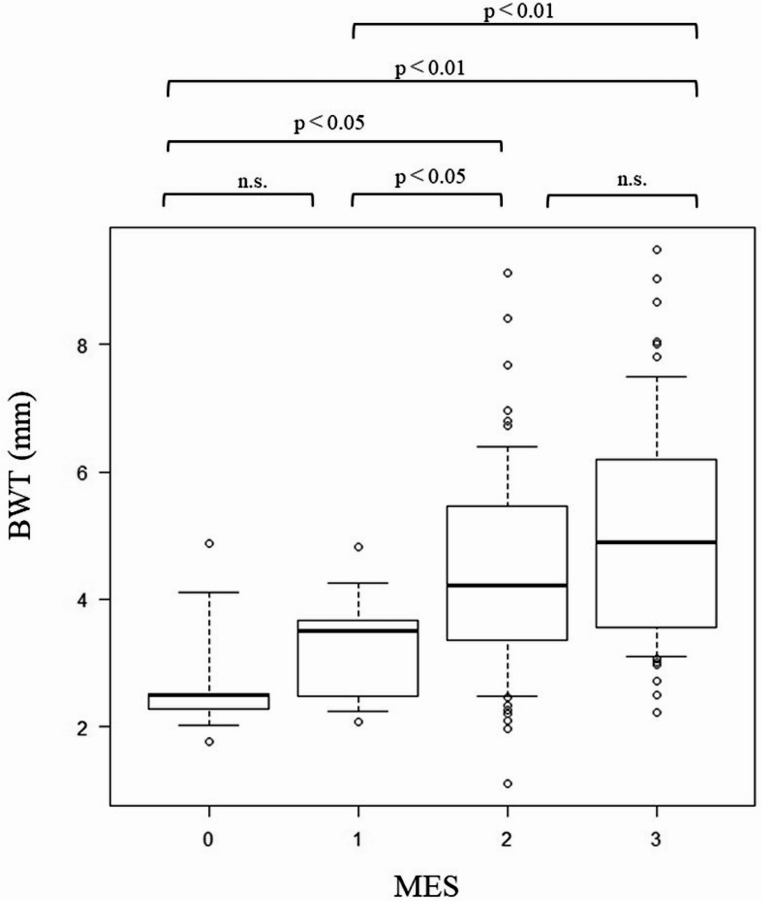
Relation between BWT and MES. BWT, bowel wall thickness; MES, Mayo endoscopic score

The mean BWT values were 2.83 ± 1.05 mm, 3.33 ± 0.90 mm, 4.70 ± 1.72 mm, and 5.36 ± 1.83 mm for patients with MES0 to 3, respectively. Patients with MES2 and 3 had significantly higher values than those with MES0 and 1 (*p* < 0.05, *p* < 0.01, respectively) (Fig. 3). There was no difference in BWT between MES0 and 1 or between MES2 and 3.

A correlation was found between US grade and BWT (*r* = 0.52, *p* < 0.01).

### Change of US grade and BWT from pre-treatment to clinical improvement

Clinical improvement was achieved within an average of 1 week (weeks 1–7) after initiating induction therapy. A total of 192 segments could be evaluated for US grade and BWT at baseline and at clinical improvement. There was a strong correlation between US grade and BWT at baseline and at clinical improvement, with correlation coefficients of 0.58 and 0.63, respectively.

In a median comparison of all patients, US grade and BWT at all segments decreased at clinical improvement (all *p* < 0.05). However, in a per-patient analysis, the US grade in 50% (16/32) of patients and BWT in 40.6% (13/32) of patients decreased only on the oral side rather than the most inflamed segments or anal side at clinical improvement.

If a decline of one grade or more is considered a US grade decline, and a decrease in BWT of 0.1 mm or more is defined as a decrease in BWT, the number of patients on the oral side, the most inflamed segments, or the anal side at the time of clinical improvement is shown in Table 2. The proportions (%) of patients with a decrease in BWT compared to those with a decline in US grade were 93.8% vs. 65.6%, 75.0% vs. 43.8%, and 65.6% vs. 46.9% on the oral side, the most severely inflamed segments, and the anal side, respectively, indicating that BWT showed a greater reduction than the US grade. The percentages of patients with a reduction in BWT and/or US grade were 96.9%, 78.1%, and 81.3% on the oral side, the most inflamed segments, and the anal side, respectively.

**Table 2:** Number of patients with reduction in US grade or BWT at clinical improvement

		Decreased US grade ≧ 1 grade					
		Oral side		Most inflamed segments		Anal side	
		Yes	No	Yes	No	Yes	No
Decreased BWT ≧ 0.1 mm	Yes	20	10	13	11	10	11
	No	1	1	1	7	5	6

US, ultrasound; BWT, bowel wall thickness

**Fig. 4:**
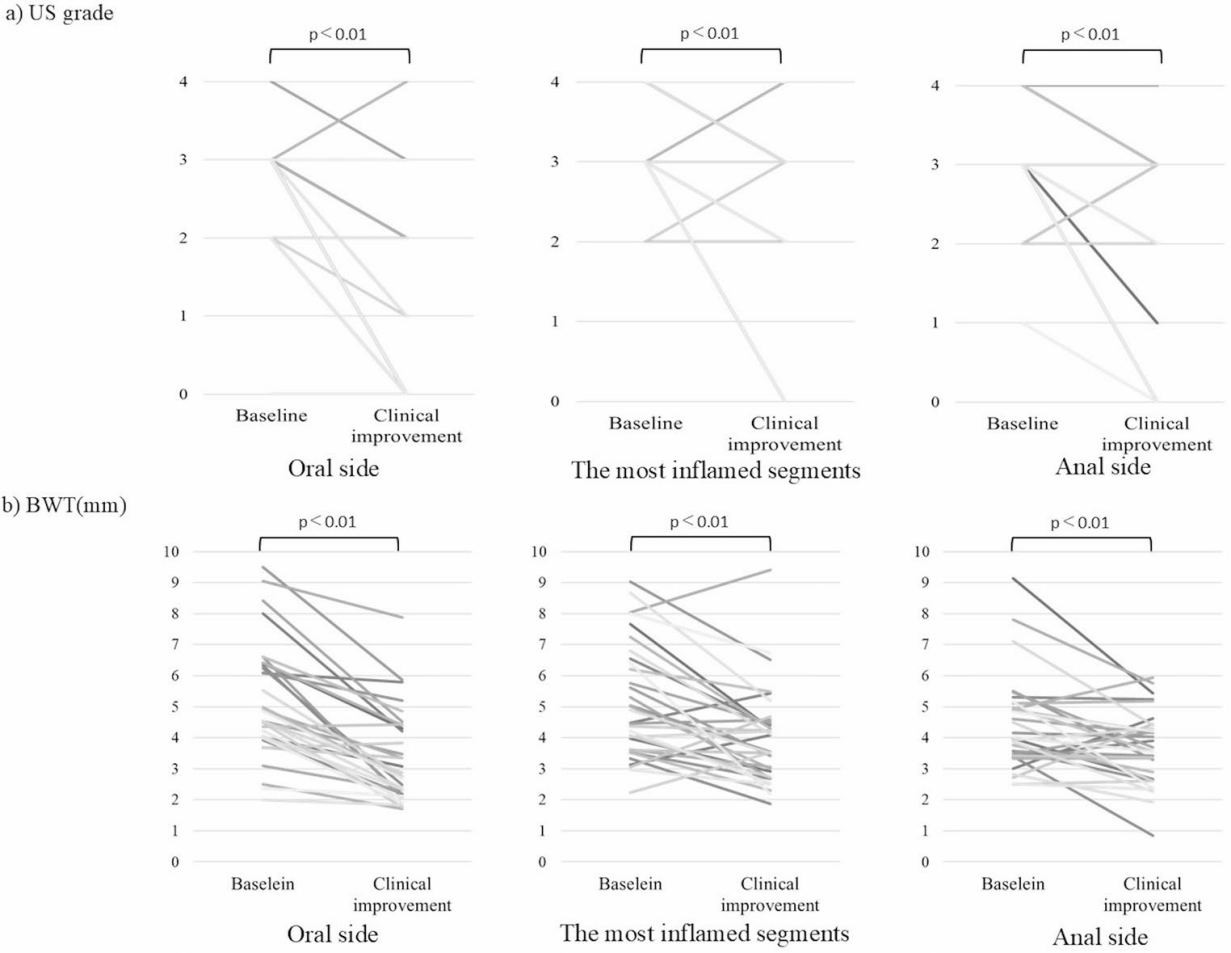
Changes in US grade and BWT from baseline to clinical improvement for the oral side, most severely inflamed segments, and anal side **a** US grade. **b** BWT (mm). US, ultrasound; BWT, bowel wall thickness

**Fig. 5:**
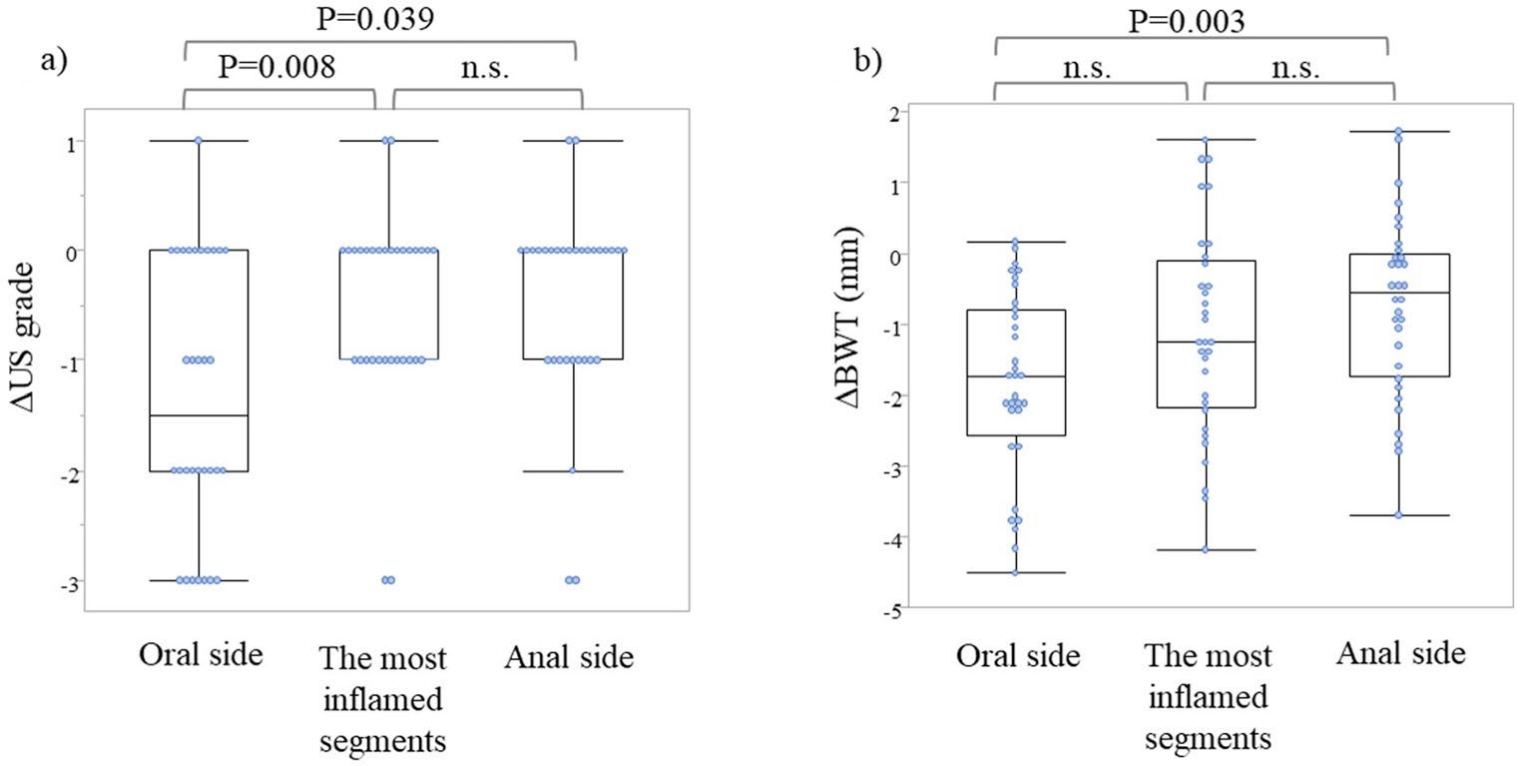
Magnitude of change in US grade and BWT for the oral side, most severely inflamed segments, and anal side. **a** US grade. **b** BWT (mm). US, ultrasound; BWT, bowel wall thickness

Changes in US grade and BWT from pre-treatment to clinical improvement are shown in Figs. 4 and 5 for the oral side, most inflamed segments, and anal side. The US grade exhibited a significant decrease from 2.53 to 1.19, from 3.25 to 2.75, and from 3.0 to 2.38 at the oral side, most inflamed segments, and anal side, respectively (*p* < 0.01). The changes in US grade were 1.34, 0.50, and 0.63 for the oral side, most inflamed segments, and anal side, respectively. The change was significantly greater on the oral side than on the most inflamed segments or the anal side (*p* = 0.008 and *p* = 0.039, respectively).

The BWT significantly decreased from 5.19 mm to 3.34 mm, from 5.15 mm to 4.0 mm, and from 4.4 mm to 3.67 mm at the oral side, most inflamed segments, and anal side, respectively (*p* < 0.01). The BWT had changed by 1.85 mm, 1.15 mm, and 0.73 mm, with reduction rates of 35.6%, 22.3%, and 16.6%, respectively, for the oral side, most inflamed segments, and anal side, respectively. Regarding the amount of change in BWT by site, the oral side did not show a significant difference from the most inflamed segments (*p* = 0.148). However, the change on the oral side was significantly greater than that on the anal side (*p* = 0.003).

## Discussion

IUS is useful for evaluating the disease status of UC in the acute stage, and its visualization rate has been reported to be higher than that of CS for all parts of the colon, except the rectum [9, 15]. In this study, the entire colon except the lower rectum could be evaluated in the acute phase using IUS. This study explored the utility of IUS for evaluating the efficacy of induction therapy in the treatment of moderate-to-severe UC. Both US grade and BWT correlated with endoscopic activity prior to the initiation of induction therapy. Furthermore, both parameters indicated that evaluation of the therapeutic effect of induction therapy should be conducted on the oral side of the colon rather than just at the most inflamed segments.

The IUS classification reported by Kinoshita et al. [9] is a four-level classification that combines BWT and changes in the layer structure of the intestinal wall, as follows: Grade 1: no thickening of the BWT; Grade 2: thickening of the mucosal layer and submucosa without hypoechoic changes in the submucosa; Grade 3: the bowel wall is thickened and the layer structure is unclear; and finally, in Grade 4: the bowel wall is thickened, the layer structure is unclear, and the mucosal surface is irregular. However, the US grade used in this study was subdivided from the perspective of changes in the layer structure of the intestinal wall. We defined US grade 0 as no wall thickening and presence of a normal layer structure, and US grade 1 as thickening of the second layer, the mucosal layer. The findings were classified into five grades. In UC, the relapse rate differs between those with MES0 and 1 [16], making it crucial to differentiate between these grades. Therefore, we subdivided the evaluation into mild UC groups. A separate, future study will clarify the usefulness of our US grade in mild UC cases. In evaluating moderate-to-severe UC, which was the subject of this study, our classification system and that of Kinoshita et al. [9] yielded nearly identical results. Our US classification correlated well with MES in active UC before induction therapy.

BWT serves as a reflection of the endoscopic findings in UC. It is characterized by high reliability and can be evaluated by technicians irrespective of their US examination experience [7, 11, 17–20]. Our US classification correlated well with BWT in active UC before induction therapy and at clinical improvement. A BWT of 3 mm was defined as the presence of intestinal activity [17, 21–24]. In this study, the initial IUS evaluation before induction therapy showed wall thickening, with the BWT measuring 3 mm or more in all patients. When comparing BWT and US grade at the time of clinical improvement, the proportion of patients with reduced BWT was higher for all regions: the oral side, most inflamed segments, and anal side.

In this study, limiting the number of US physicians to one may have contributed to the favorable outcomes in BWT. In cases of UC with repeated flare-ups, the colonic walls become stiffer, making their thickness less susceptible to changes in the degree of inflammation. The low number of relapses included in this study may have contributed to the strong performance of BWT. This study revealed no difference in BWT between MES0 and 1 and between MES2 and 3, consistent with previous studies [21]. BWT may be inappropriate to detect the presence of mild inflammation or distinguish between different states of severe inflammation. Furthermore, the change in BWT differed from that of the change in US grade from pre-treatment to clinical improvement. Compared to the changes in the titer of BWT at the most inflamed segments, those on the oral side did not reach statistical significance.

The purpose of this study was to assess the usefulness of IDUS in induction therapy, particularly by identifying appropriate evaluation sites, rather than comparing the efficacy of BWT and US grade. In addition, the study design did not allow for consideration of the specificity of BWT and US grades. The combination of BWT and US grades can be used to evaluate the effectiveness of induction therapy more appropriately on the oral side than at the most inflamed site. The US grade can objectively record echo changes in the colon wall, reflecting the degree of inflammation. Our US classification enables the ongoing evaluation of mucosal healing following clinical improvement achieved through induction therapy. However, further research is needed to validate this aspect in the future.

A previous study demonstrated that incorporating an assessment of the Doppler signal increase and loss in bowel wall stratification enhanced the positive likelihood ratio for the identification of inflammatory lesions [15]. Evaluation of intestinal blood flow using IUS is useful for determining the activity of IBD patients [23, 25], but it is influenced by various factors, such as the type of diagnostic device and the patient’s physique [9]. Cases in which hyper-flow is observed are useful for evaluating induction therapy, but there are also issues, such as patients with no hyper-flow in active UC. The utility of incorporating blood flow information into BWT to determine the therapeutic efficacy of induction therapy for UC needs to be investigated in future studies.

Few studies have explored the utility of IUS in assessing the treatment efficacy for active UC. BWT can serve as an indicator of the efficacy of steroid treatment [3, 25], the necessity for surgery after steroid treatment [5], and the impact of tofacitinib treatment [20]. Previous reports have not explored the different regions of the colon. In contrast, the present study systematically assessed three specific sites over a defined period: the oral side, the most inflamed segments, and the anal side, from pre-treatment to clinical improvement.

It has been previously demonstrated that complete histological normalization [26, 27] is associated with improved clinical outcomes. However, when the type of normalization was categorized into partial (including proximal, distal, and patchy histological normalization) and complete sub-groups, only patients who achieved complete histological normalization exhibited a reduced risk of clinical relapse [10]. We could not determine whether the narrowing of the inflamed segments was predictive of reducing clinical relapse in the long term, but we demonstrated that IUS was useful in predicting the effects of induction therapy in this study.

Our study had some limitations. First, this was a retrospective study conducted by a single IUS practitioner at a single institution. Second, a CS evaluation was not possible at clinical improvement. Third, blood flow was not considered when assessing activity using IUS. Fourth, few patients enrolled in this study had repeated flare-ups.

## Conclusion

In patients with active UC, our US grade classification and BWT evaluation using IUS well correlated with MES. IUS is effective for noninvasively assessing the efficacy of induction therapy and should be performed at the more proximal part of the colon as opposed to solely at the site of the most severe inflammation.

## Electronic supplementary material

Below is the link to the electronic supplementary material.

Ultrasound (US) grade classification a) US grade 0: normal thickness of all colonic wall layers b) US grade 1: thickening of the second layer (mucosa) (arrows) c) US grade 2: thickening of second and third layers (submucosa) without hypoechoic change in the third layer (arrowheads) d) US grade 3: thickening of second and third layers with hypoechoic changes in the third layer e) US grade 4: bowel wall thickness with loss of stratification and irregular mucosa Supplementary Material 1

Bowel wall thickness (BWT). BWT measurement: The vertical distance from the mucosal border echo (arrows) to the serosal border echo (arrowheads) within the intestinal wall was assessed at three distinct locations (sites a–c), and the resultant average value was used Supplementary Material 2

## Data Availability

The datasets generated and/or analyzed during the current study are available from the corresponding author on reasonable request.
